# Incarcerated Gravid Uterus: Spontaneous Resolution Is Not Rare

**DOI:** 10.3390/diagnostics11091544

**Published:** 2021-08-25

**Authors:** Daisuke Tachibana, Takuya Misugi, Kohei Kitada, Yasushi Kurihara, Mie Tahara, Akihiro Hamuro, Akemi Nakano, Akira Yamamoto, Masayasu Koyama

**Affiliations:** 1 Department of Obstetrics and Gynecology, Osaka City University Graduate School of Medicine, Osaka 545-8585, Japan; misutaku1975@infoseek.jp (T.M.); kafukafu0404@yahoo.co.jp (K.K.); kurikuri_1011@yahoo.co.jp (Y.K.); rxv13436@nifty.ne.jp (M.T.); hamuroa@med.osaka-cu.ac.jp (A.H.); m2037746@med.osaka-cu.ac.jp (A.N.); masayasukoyama@gmail.com (M.K.); 2 Department of Diagnostic and Interventional Radiology, Osaka City University Graduate School of Medicine, Osaka 545-8585, Japan; loveakirayamamoto@gmail.com

**Keywords:** incarcerated gravid uterus, spontaneous resolution, manual reduction, diagnosis, management

## Abstract

Aim: Incarcerated gravid uterus is a rare obstetrical complication that leads to adverse outcomes, especially if the uterus remains incarcerated and the condition goes undiagnosed until delivery. However, there is no consensus regarding the optimal management of this complication because of its rarity. In this study, we aimed to elucidate the incidence of incarcerated gravid uterus, as well as its natural courses and perinatal outcomes. Methods: We retrospectively reviewed medical records of patients who had incarcerated gravid uterus and managed at Osaka City University Hospital between April 2011 and March 2021. Incarcerated gravid uterus was defined as a retroverted or retroflexed uterus after 16 weeks of gestation. Results: There were 14 incarcerated cases among 6958 pregnant women, and 13 of them had some kind of gynecological complication and/or history. Spontaneous resolution of incarcerated gravid uterus after 16 gestational weeks was observed in six cases before the late second trimester and five cases at the late second trimester to early third trimester. Three cases remained incarcerated at term or near-term. One case with adenomyosis had severe abdominal pain, although it was difficult to ascertain whether the cause of pain was triggered by adenomyosis and/or incarceration. One case was misdiagnosed as placenta previa, and the uterine cervix was subsequently injured during cesarean delivery, resulting in massive hemorrhaging. Conclusions: Approximately 1 in 2300 pregnancies continued to be in an incarcerated condition at term or near-term, and 78.5% of cases showed a spontaneous resolution after 16 weeks of gestation. Expectant management with careful attention to the incarcerated gravid uterus may be one option in situations where there are no severe symptoms related to the incarceration itself.

## 1. Introduction

Uterine retroversion or retroflex is observed in 15% of pregnant women during the first trimester, and a majority will spontaneously resolve before 14 weeks of gestation [1]. If the uterine fundus remains in the pelvic cavity without self-correction after 16 weeks, the condition is said to be incarcerated. Incarcerated gravid uterus is a rare condition, occurring in approximately 1 in 3000 pregnancies [2]. Proposed risk factors are endometriosis, pelvic inflammatory disease, previous abdominal or pelvic surgery, fibroid, uterine anomaly, and uterine retroversion prior to pregnancy [1,2]. Symptoms of uterine incarceration during pregnancy are thought to be non-specific, such as pelvic discomfort, urinary retention, and gastrointestinal symptoms, and some patients are asymptomatic altogether [1,3,4]. Diagnosis is sometimes difficult because the hints of this disease are quite ambiguous, such as a non-palpable cervix and/or pelvic mass in filling the posterior cul-de-sac upon vaginal examination [1,2]. If a gravid uterus remains to be incarcerated, adverse outcomes, such as urinary retention, renal failure, miscarriage, preterm labor, and thrombosis, can be anticipated [3,5,6,7,8].

Some authors suggest the use of manual reduction for the incarcerated uterus after 16 weeks of gestation. However, these procedures require hospitalization and invasive stress, including anesthesia and even laparotomy [1,2,5,8]. Moreover, because incarceration itself potentially implies uterine adhesion to surrounding pelvic organs, which is difficult correctly judge by magnetic resonance imaging (MRI) and/or ultrasound during pregnancy, manual reduction itself may cause potentially severe complications [6,9,10,11]. As such, obstetricians may face the dilemma of deciding whether to perform the manual reduction or not, and the selection of cases suitable for reduction itself is quite difficult prior to the procedure.

In this study, we aimed to elucidate the incidence, natural courses, and perinatal outcomes for incarcerated gravid uterus.

## 2. Methods

### 2.1. Study Design, Ethical Approval, and Study Population

The medical records of pregnant women who had incarcerated gravid uterus and who delivered at Osaka City University Hospital between April 2011 and March 2021 were retrospectively reviewed. All patients gave their informed written consent, and the study protocol was approved by the institutional review board (No. 2021-067, May 2021).

### 2.2. Definition of Incarcerated Gravid Uterus and Data Collection

Incarcerated gravid uterus was defined as a retroverted or retroflexed uterus after 16 weeks of gestation. We obtained medical information as follows: age, gravida/parity, mode of conception, gynecological complication and history, diagnosed week of incarceration, weeks of estimated resolution, history of characteristic pregnancy course, and delivery outcomes. The estimated gestational week of resolution was indicated by the duration between the gestational week of the last recognition of an incarcerated uterus and the gestational week of the confirmation of resolution. The resolution was confirmed by vaginal examination, MRI, and/or ultrasound evaluation.

## 3. Results

During the observational period, there were 6958 deliveries at Osaka City University Hospital, and no cases were referred to our hospital with the diagnosis of incarcerated gravid uterus. Incarcerated gravid uterus after 16 gestational weeks was diagnosed in 14 cases, and spontaneous resolution was recognized in 11 cases. The incidence of incarcerated gravid uterus after 16 gestational weeks and spontaneous resolution in the observed period were 0.2% (14/6958) and 78.5% (11/14), respectively. In all of the cases except for one, which was not diagnosed until cesarean delivery, the initial finding of suspected uterine incarceration was a non-palpable and non-visualized cervix upon vaginal examination.

Table 1 shows a summary of the cases. Thirteen women among them had some gynecological complication, such as fibroids, adenomyosis, history of peritonitis, endometriosis, and tubal pregnancy. Case 8 experienced abdominal pain that continued for about 4 h, potentially at the timing of spontaneous resolution and around 28 weeks of gestation.

**Figure 1:**
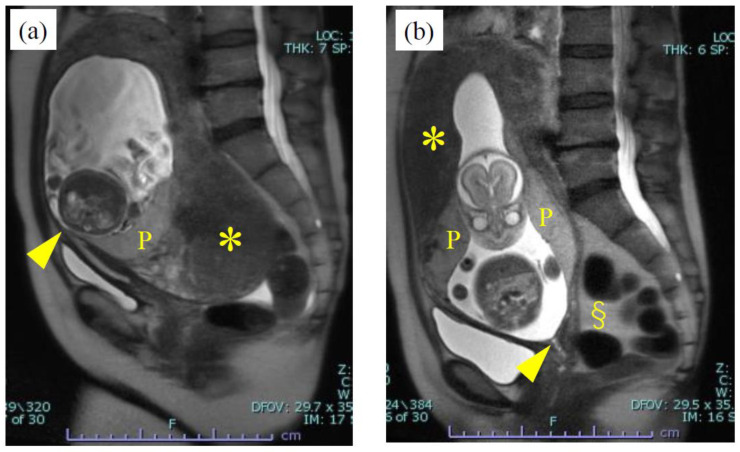
Sagittal T2-weighted MR images of Case 2. An enlarged uterine fundus with adenomyosis was entrapped within the pelvic cavity, and the cervix was stretched cranioventrally at 20 weeks of gestation (**a**). However, at 21 weeks, spontaneous resolution was confirmed with the finding of a normal position of the cervix (**b**). The arrowhead indicates cervical internal ostium. P, placenta; §, the amount of ascites was relatively larger than the normal physiological amount during pregnancy; * adenomyosis.

We describe three cases in detail, all of which showed characteristic findings and pregnancy courses due to incarceration. Case 2, who was complicated with adenomyosis, was transferred to our hospital due to severe abdominal pain with elevated inflammation signs at 20 weeks of gestation (white blood cell count: 15,000/μL; C-reactive protein: 12.7 mg/dL), and tramadol hydrochloride, acetaminophen, and pentazocine hydrochloride were needed to control her abdominal pain. The incarcerated uterus was identified by MRI, and was found to have an enlarged fundus with adenomyosis (Figure 1a). Antibiotic administration was not effective and was discontinued after five days. However, the patient was slightly relieved from severe pain at 21 weeks; MRI revealed a spontaneous resolution of the incarcerated uterus, and the lesion of adenomyosis was recognized as somehow reduced from its fully swollen size (Figure 1b). Despite the uterine resolution, the patient intermittently needed painkillers. Cesarean delivery was performed with the diagnosis of breech presentation and labor onset at 29 weeks of gestation.

**Figure 2:**
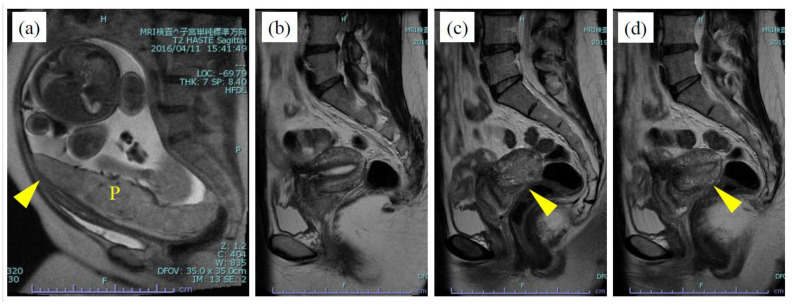
Sagittal T2-weighted MR images of Case 13. They showed placenta previa at the cranioventrally stretched internal cervical ostium (arrowhead) at 33 weeks of gestation (**a**). MRI after the delivery showed signs of severe adhesion (**b**); retroflexed uterus (**c**); elevated posterior vaginal fornix (**d**); loss of the fatty layer at the posterior cul-de-sac between the posterior uterine wall and the sigmoid colon (**b**–**d**). P, placenta. MRI imaging of the uterus without using contrast agent.

Case 13, conceived by assisted reproductive technology, was strongly suspected to have severe adhesion of the Douglas’s pouch as a result of a previous history of peritonitis, an operation for endometrial ovarian cysts, and a tubal pregnancy. MRI showed placenta previa at the cranioventrally stretched internal cervical ostium at 33 weeks of gestation (Figure 2a); however, it was difficult to diagnose the adhesion behind the uterine cavity. A cesarean delivery was performed because of increased uterine contractions at 34 weeks of gestation. MRI after the delivery showed signs of adhesion [10], a retroflexed uterus, an elevated posterior vaginal fornix, and loss of the fatty layer at the posterior cul-de-sac between the posterior uterine wall and the rectum (Figure 2b–d).

Case 14 had undergone cesarean delivery with a misdiagnosis of placental previa without the prior recognition of incarceration. As a result, the uterine cervix was incised by 3/4 of its total circumference, and the baby was born through the posterior uterine wall. Although the estimated blood loss amounted to 4.8 L, the amputated lesion of the cervix was successfully repaired by one-layer single knot suturing using 1-0 Vicryl, and the post-operative course was uneventful. This patient experienced two subsequent spontaneous pregnancies that resulted in uncomplicated pregnant courses, and cesarean deliveries were performed at term without incarceration.

The other 11 cases did not experience any severe abdominal pain, although they did complain about the frequency of urination and abdominal discomfort, which were thought to be within physiological ranges and did not necessitate any medical treatment.

## 4. Discussion

Our study demonstrated that roughly one pregnancy in 2300 deliveries remained complicated with incarcerated uterus, at term or near term, if manual reduction was not performed. In addition, an antenatal diagnosis was correctly made in 92.8% of cases, and spontaneous resolution after 16 gestational weeks was observed in 78.5%. However, severe operative injury was experienced in one case misdiagnosed as placenta previa. As far as we know, our study is the first to include such a large number of patients and to report on the natural courses of incarcerated gravid uterus after 16 weeks of gestation without any manual reduction.

Complications that arise from an incarcerated uterus are thought to result in adverse perinatal outcomes [1,2,3,5,6,7,8]. Extreme dislocation of the uterine cervix causes compression of the urethra and anatomical distortion of the bladder, thus presenting symptoms like dysuria, urinary retention, and overflow incontinence, as well as abdominal pain and worsening constipation [1,2,8]. Reportedly, the most severe cases led to bladder atony, renal failure, hypertensive disorders of pregnancy, and pulmonary embolism [5,7,8]. For these reasons, some authors recommend attempting the reduction of the incarcerated uterus without any delay [8,12,13]. However, there is no consensus as to the optimal management of this complication due to its rarity.

Four cases of spontaneous resolution in the third trimester have been reported so far [5,14,15,16]. Their corrections of uterine incarceration were observed between 30 and 36 weeks, and two cases among them were complicated with fibroids. Interestingly, three cases in these reports experienced resolution without any abdominal pain, and were incidentally diagnosed as corrected. Moreover, abdominal and micturition symptoms disappeared without any treatment as the gestation progressed, even while the uterus remained incarcerated. Regarding this phenomenon, Smalbraak et al. speculated that bladder symptoms may improve when the anterior wall of the uterus becomes thinly stretched in order to accommodate the growing fetus, and this condition is the so-called “uterine sacculation” [5,17]. However, presently, it is impossible to predict who has a higher chance for spontaneous resolution. In addition, it is also difficult to assume who is a candidate for manual reduction because of possible severe symptoms if the incarceration continues. In fact, as we showed in Case 13, it is difficult to evaluate the adhesion behind the uterine cavity during pregnancy. Furthermore, obstetricians should keep in mind that manual reduction may cause serious complications, possibly resulting in the rupture of membranes and intrauterine fetal death [6,9,10,11]. Taken together with these facts, intervention should be reserved for only highly symptomatic cases, and expectant management should be the standard of care. We also suggest a redefinition of uterine incarceration, since this term should be reserved for symptomatic cases, as asymptomatic cases seem to be variations of a normal anatomy.

The limitations of this study are the retrospective nature of the study design and the fact that the number of patients was too small to elucidate concrete evidence. Furthermore, we did not validate the efficacy of manual and/or passive reduction of the incarcerated gravid uterus [18]. However, as far as we know, this is the largest case series in the past three decades where diagnostic tools, including MRI and ultrasound, became prevalent in clinical practice to precisely diagnose incarceration of the uterus during pregnancy.

In conclusion, the first finding to suspect incarcerated gravid uterus is the difficulty in recognizing the uterine cervix by vaginal examination. For such cases, MRI and ultrasound should be undertaken to confirm the diagnosis. In cases where there are no severe symptoms, expectant management with careful monitoring might be an option. Our findings will provide helpful information for obstetricians in clinical practice.

## Figures and Tables

**Table 1:** Characteristics of the patients.

Case	Age	G/P	Mode of Conception	Gynecological Complication	Diagnosis (Gestational Week)	Resolution (Gestational Week)	Outcome
1	36	1/0	spontaneous	fibroid (6 cm)	16	16 to 20	VD at 38 weeks
2	41	2/0	spontaneous	adenomyosis	20 (severe pain)	20 to 21	CS at 29 weeks (labor onset, breech presentation)
3	37	2/0	spontaneous	fibroid (11 cm)	20	20 to 21	CS at 38 weeks (labor arrest)
4	33	2/0	spontaneous	fibroid (9 cm)	16	21 to 23	VD at 40 weeks
5	33	1/0	spontaneous	fibroid (7 cm)	16	16 to 24	VD at 41 weeks
6	31	1/0	spontaneous	fibroid (7 cm)	16	24 to 26	VD at 40 weeks
7	36	2/0	ART	none	16	26 to 28	VD at 39 weeks
8	36	2/0	ART	fibroid (4 cm)	26	28 to 29	VD at 37 weeks
9	40	2/0	spontaneous	fibroid (9 cm)	19	27 to 29	CS at 36 weeks (breech presentation)
10	41	1/0	spontaneous	fibroid (7 cm)	20	29 to 30	CS at 38 weeks (labor arrest)
11	42	1/0	spontaneous	fibroid (13 cm)	20	29 to 31	CS at 37 weeks (breech presentation)
12	35	1/0	spontaneous	fibroid (6 cm)	37	Not resolved	CS at 38 weeks
13	41	1/0	ART	Peritonitis, ovarian endometrial cyst, and tubal pregnancy	31	Not resolved	CS at 34 weeks
14	36	2/0	spontaneous	fibroid (7 cm)	Misdiagnosed as placenta previa	Not resolved	CS at 37 weeks

G/P: gravida/para, ART: assisted reproductive technology, GW: gestational week, VD: vaginal delivery, CS: cesarean section, at: at.

## Data Availability

Data available on request due to restrictions of privacy.
